# Molecular assessment of visitor personal protective equipment contamination with the Aleutian mink disease virus and porcine circovirus-2 in mink and porcine farms

**DOI:** 10.1371/journal.pone.0203144

**Published:** 2018-08-27

**Authors:** José Manuel Díaz Cao, Alberto Prieto, Gonzalo López, Ricardo Fernández-Antonio, Pablo Díaz, Ceferino López, Susana Remesar, Pablo Díez-Baños, Gonzalo Fernández

**Affiliations:** 1 Department of Animal Pathology (INVESAGA Group), Faculty of Veterinary Sciences, Universidade de Santiago de Compostela, Lugo, Spain; 2 Galician Association of Mink Breeders (AGAVI), Santiago de Compostela, Spain; Ross University School of Veterinary Medicine, SAINT KITTS AND NEVIS

## Abstract

Personal protective equipment (PPE) is an element of biosecurity intended to prevent the access or spread of diseases in farms. Nevertheless, to date no extensive reports exist about the effectiveness of different available PPE on farms. Thus, our aim was to estimate the degree of protection of PPE from viral contamination during farm visits. Two farms, infected with Aleutian mink disease virus and porcine circovirus–2 respectively, were visited by six visitors wearing different combinations of PPE: coveralls with hood and bootcovers, both with a certified barrier to infective agents (certified PPE group) and non-certified bootcover and coverall without hood (non-certified PPE group). Seventy-two swab samples from PPE and both hair and street clothes under PPE were taken after the visit and analysed by qPCR. Our results reveal viral exposure during visits, and the external protections of body and shoes were contaminated in all cases (24/24). In addition, protection from viral contamination varied noticeably according to the biosecurity elements used. A higher number of positives were detected in the non-certified PPE group than in the certified PPE group, both in elements under external protections (14/18 vs 3/18) and also in hair (4/6 vs 0/6). In fact, non-certified bootcovers broke during visits, resulting in viral contamination of the internal elements under them; these are consequently not suitable for using with wrinkled surfaces usually found in farm facilities. Thus, certified coveralls should be used in order to prevent contaminations, and workers and personnel of farms should be trained in their proper use. qPCR is a useful tool in the risk management of biosecurity programmes, and our results may serve as a model to evaluate different biosecurity measures.

## Introduction

Biosecurity is defined as management activities that reduce the opportunities for infectious agents to gain access to, or spread within, a production unit [1]. The implementation of such measures constitutes an important element for disease prevention in farms. Therefore, a correct application of biosecurity measures has proved to improve health and productivity, as well as decrease the need for administration of antimicrobial drugs [2].

A number of different measures can be taken, but their suitability to control a disease is determined by the epidemiological characteristics of pathogens. In the case of agents with ability to persist for a long time in the environment, such as the Aleutian mink disease virus (AMDV; Carnivore amdoparvovirus– 1) or porcine circovirus–2 (PCV-2), indirect contact requires special attention to the diffusion of the pathogen [3–6]. Indirect transmission of diseases involves different elements. Among them, vehicles, equipment, and persons are identified as moveable risks, and they constitute an important indirect route for the spread of several contagious livestock diseases between herds [7–9]. Thus, people entering the farm have been recognized as a major pathway of disease introduction during outbreaks of highly contagious diseases [10]. Additionally, contaminated people are one of the main causes of farm reinfections, and consequently, they are a cause of failure of eradication programmes in some diseases such as Aleutian mink disease [11]. Strategies to prevent this situation usually imply visitors using boots or clothes supplied by the farm or the use of disposable personal protective equipment (PPE), such as coveralls or bootcovers that are removed after the visit. Measures are not standardized in production farms and may vary significantly from one farm to another; the validity of some types of equipment in preventing contaminations is also limited [12]. Moreover, PPE items are potential fomites and may play a role in the transmission of disease if they become contaminated with infectious microorganisms; however this remains as a poorly understood area in need of research [13]. Furthermore, there are no reports of effective protocols of use of PPE for visitors to infected farms in order to minimize the risks of contamination.

qPCR can be a valuable tool in the detection and quantification of viral contamination in order to determine risks and optimize biosecurity measures. Measuring viruses on surfaces is important for understanding the distribution of infectious agents in the environment and assessing the role of fomites in disease transmission [14]. Thus, it has been possible to prove the contamination of fomites, including PPE, by viral species such as influenza or porcine reproductive and respiratory syndrome virus in porcine farms [15–17] or by AMDV in mink farms [18,19]. Actually, the presence of humans on the AMDV-positive farms for 30 min was sufficient for qPCR detection of viral DNA in coveralls and bootcovers [18]. Thus, the evaluation of the protection provided by PPE and the design of effective protocols of use seems critical to avoid contamination of visitors that can contribute to the dissemination of viruses to other farms.

The aim of this study was to assess by qPCR the contamination of PPE during farm visits and the degree of protection they conferred by using as model two different viruses (AMDV and PCV-2).

## Material and methods

### Ethics statement

We declare that this study did not required specific permissions because none of the diseases considered in this study are mandatory for notification in Spain or in European Union and legal measures are not required in farms where these diseases are detected. The owners of the farms gave permission to conduct the study on these farms. Field studies did not involve endangered or protected species.

### Included farms, sampling method and sample categorization

The study was performed on two farms located in Galicia (NW Spain): a mink farm that had remained infected with AMDV since 2012, with a seroprevalence of 25.65% by counterimmunoelectrophoresis and no eradication measures applied; and a PCV-2-positive porcine farm, showing infected animals by qPCR in 2017. Vaccination was not performed against PCV-2.

On each farm, six visitors entered at the same time and wandered around the facilities. Visitors were divided in two groups: certified PPE group and non-certified PPE group; according to the combination of PPE they wore (Table 1).

**Table 1:** Samples taken of each element of biosecurity prior and after visit.

Elements of biosecurity (from external to internal)	2 farms (AMDV-positive and PCV-2-positive) (6 visitors per farm)			
	Certified PPE group (3 visitors per farm)		Non-certified PPE group (3 visitors per farm)	
	Samples taken prior visit	Samples taken after visit	Samples taken prior visit	Samples taken after visit
	3 samples per visitor	6 samples per visitor	2 samples per visitor	6 samples per visitor
	Samples per farm (27)		Samples per farm (24)	
Body:				
Simple coverall (S-Coverall)	N.W.	N.W.		S
Certified coveralls (C-Coverall)		S		S
Street clothes	S	S		
Shoes				
Certified bootcovers (C-Bootcovers)		S	N.W.	N.W
Simple bootcovers (S-Bootcovers)	N.W.	N.W.		S
Shoe covers		S		S
Street shoes	S	S	S	S
Hood:	Hood on		No hood	
Hair	S	S	S	S

S: Sampled, N.W. Not worn in this group

The certified PPE group was used to test certified PPE. Visitors wore certified coveralls (C-Coverall; Biztex® Microporous 6/5 Coverall, Portwest Ltd, Ireland) conforming to the standards EN 14126 (barrier to infective agents), EN ISO 13982–1, and EN 13034 (barrier against airborne dry particulates and liquid chemicals), and they wore the hood. They also wore certified bootcovers (C-Bootcovers; Tychem® C, model POBA, DuPont, USA), conforming to EN 14126 (barrier against infective agents), EN 863 (puncture resistance), and DIN EN ISO 13934–1 (resistant to traction), over non-certified simple polypropylene shoe covers.

The non-certified PPE group was used to test non-certified PPE, and visitors wore non-certified polypropylene coveralls (S-Coveralls: simple coveralls) over certified coveralls and they did not wear the hood. They also wore non-certified polyethylene bootcovers (S-Bootcovers: simple bootcovers) over non-certified simple shoe covers. Clothing models for certified and non-certified PPE groups are provided in S1 File.

Hair, street shoes, and clothes of visitors were tested as control prior to their entrance on the farms and dressing with PPE (Table 1). Visitors also wore disposable gloves to avoid contamination of hands. The visit consisted of wandering within farms’ barns/rooms for 60 minutes. As a part of the visit, environmental swabs were additionally taken from different surfaces of the farm in order to determine the presence of environmental viral DNA. Both PPE and environmental samples were collected by swabbing each area with a dry sterile cotton swab (11 mm in diameter); then the swab head was placed in a sterile tube. Samples after each visit were taken outside the farm; the integrity of PPE was assessed prior to sample collection. Each swab sample for street shoes, bootcovers, and shoecovers was taken by swabbing 5 times all of the surfaces of soles; C-Coverall, S-Coverall, and street clothes were sampled by swabbing 5 times each arm, leg, and the anterior trunk of the body. Finally, hair was sampled by swabbing 10 times. The order of sampling was hair, C-Bootcovers (certified PPE group) or S-Bootcovers (non-certified PPE group), shoe covers, street shoes, and then C-Coverall and street clothes (certified PPE group) or C-Coverall and S-Coverall (non-certified PPE group). Environmental samples on the mink farm were collected from cages (n = 3) and corridors within barns (n = 3), swabbing 5 times the walls and floors. On the porcine farm, floor (n = 3) and wall samples (n = 3), from animal pens were collected swabbing along a distance of 4 meters. Finally, a floor sample was taken outside the farm, where PPE samples were collected, to test whether this place was contaminated. After the visit, all the PPE used in this study were introduced in a container with hermetic closure to be destroyed by an authorized company, following the rules of the unit of waste management of the Universidade de Santiago de Compostela. Visitors performed hand hygiene with an alcohol-based hand rub and water whenever ungloved hands. Street shoes soles were disinfected with bleach.

Thus, 58 samples per farm were taken (n = 116): 15 clothes/hair samples prior to the visit, 36 PPE samples after the visit, 6 surface samples on the farm, and 1 sample in the PPE sampling place. At the laboratory, samples were frozen at −20°C until processed.

### Sample preparation, DNA extraction and qPCR

Sample tubes were eluted in 5 ml of sterile phosphate-buffered saline with 0.05% Tween 20 (all reagents supplied by Sigma-Aldrich, Missouri, United States) and vortexed for 1 min. After 15 min of settling, 1 ml of supernatant from each sample was taken and placed in a sterile Eppendorf tube, and then kept at −20°C. DNA was extracted from 200 μl of supernatant using a combination of two commercial kits as previously described [18]. Briefly, all samples were firstly processed by a general DNA extraction method (High Pure PCR Template Preparation Kit, Roche Diagnostics GmbH, Mannheim, Germany) and analysed by qPCR. Subsequently, negative samples were retested after a new extraction with a specific procedure for soil samples (Nucleospin® Soil, Macherey-Nagel GmbH & Co KG, Düren, Germany). An internal control of synthetic DNA was included in each extraction to identify possible qPCR inhibitions.

qPCR analysis was run on an Applied Biosystems ABI Prism 7500 thermocycler (Thermo Fisher Scientific, Waltham, USA). The commercial kits AMDV Genesig Advanced Real-Time PCR Detection Kit (Primerdesign Ltd, Cambridge, UK) and EXOone PCV2 oneMIX (EXOPOL SL, Zaragoza, Spain) were used for amplifying AMDV NS1 and PCV2 orf-2 genes, respectively. Positive controls provided by the manufacturers were ten-fold diluted from 2 x 10^5^ copies/μl to 2 copies/μl for the preparation of the standard curve. The quantified DNA was expressed as number of copies/sample. For both viruses, the number and percentage of positive samples, mean of copies, standard error, and range were determined from qPCR results for each sample category.

## Results

qPCR standard curves were y = 37.789–3.422x and y = 42.11–3.52x for AMDV and PCV2, respectively. In both cases, the standard curves showed a very good fit (R^2^ = 0.999). In general, both environmental and PPE samples from the AMDV-positive farm presented higher values for mean virus copies per sample.

All samples taken before the visits were negative for both viruses (n = 30). After the visit, contaminated PPE was detected in both groups and for both pathogens, resulting in a total of 45/72 (62.5%) positive samples. In general, a higher number of positives was detected in group B for both farms (Table 2). The most external protections of body and shoes were contaminated in all cases, but the degree of contamination of protections covered by other elements of protection was variable (Table 2). In the certified PPE group, 2/6 of the samples of street clothes tested positive (one in each farm, involving a different visitor), but in the non-certified PPE group, contamination was detected in 3/6 internal C-Coverall (2/3 positive samples in the AMDV-positive farm and 1/3 positive samples in the PCV-2-positive farm), despite the fact that S-Coverall were worn externally. No signs of rupture were observed in any of the coveralls. No positive samples of street shoes and 1/6 of shoe covers were detected in the certified PPE group; in contrast, most street shoes and shoe covers from the non-certified PPE group were positive (Table 2). All S-Bootcovers of the non-certified PPE group and 1/3 of C-Bootcovers of the certified-PPE group presented gashes. Broken C-Bootcovers in the certified-PPE group coincided with the positive shoe covers. Hair samples from visitors from certified group (who wore the hood), were negative, but hair from visitors from the non-certified PPE group (without the hood) presented 4/6 positive samples (3/3 positive samples in the AMDV-positive farm and 1/3 positive samples in the PCV-2-positive farm).

**Table 2:** Results of qPCR from personal protective equipments after the visit to positive farms to AMDV and PCV-2.

AMDV-positive farm				PCV-2-positive farm		
Certified PPE group						
Element	N° positive^a^	Mean virus copies per sample (standard error)	Range	N° positive^a^	Mean virus copies per sample (standard error)	Range
C-Coverall	3	3,325 (2,263.32)	290–7,751	3	21,433 (1622)	19,621–24,669
Street clothes	1	100		1	1655	
C-Bootcovers	3	5,783 (2,074)	3,525–9,926	3	113,673 (45,258)	42,180–197,497
Shoe covers	1	139		0		
Street shoes	0			0		
Hair	0			0		
Non-certified PPE group						
Element	N° positive^a^	Mean virus copies per sample (standard error)	Range	N° positive^a^	Mean virus copies per sample (standard error)	Range
S-Coverall	3	446 (138)	216–693	3	4,913 (3,055)	827–10,890
C-Coverall	2	17 (3)	14–19	1	5,774	
S-Bootcovers	3	2,000 (348)	1,363–2,564	3	24,258 (13,646)	3,535–50,000
Shoe covers	3	26,064 (9979)	11,156–45,010	3	36,795 (20,801)	9,008–77,502
Street shoes	3	72 (60)	10–92	2	15,046 (14,006)	1,040–29,052
Hair	3	213 (156)	27–552	1	693	

C-Coverall: certified coverall; S-Coverall: simple coverall; C-Bootcovers: certified bootcovers; S-Bootcovers: simple bootcovers.

^a^ 3 samples in each category

All samples from environmental surfaces were positive (Table 3), indicating the presence of contamination with DNA of these pathogens on farms. Viral DNA was not detected in the floor samples from the site where PPE samples were taken on both farms.

**Table 3:** Results of the qPCR in the environmental surface samples.

Sample	N° positive^a^	Mean virus copies per reaction (standard error)	Range
AMDV-positive farm			
Barns floor	3	16,414 (8,199)	734–31,704
Animal cages	3	211,946 (118,541)	3,065–530.437
PCV-2-positive farm			
Pen floor	3	122,231(79,987)	17,555–279,337
Pen walls	3	40,141 (32,929)	7,212–73,070

^a^ 3 samples tested in each category

## Discussion

Knowledge of the role of personnel in the indirect transmission of viruses may be of importance for understanding the dynamics of viral infections. For example, monitoring surfaces has been shown to be essential to understand the dissemination of viruses in hospitals or day-care centers, and it has helped to establish preventive measures [20,21]. In order to determine the dynamics of virus survival and transmission via contaminated PPE and the attendant health risks, levels of viral contamination on PPE need to be quantified [13]. Although this risk has been recognised, the magnitude of risk is difficult to assess because there are few data available on levels of viral contamination on PPE after use on farms. This requires effective and reproducible methods to recover infectious viruses from items of PPE [13]. qPCR may help in this purpose; however, the presence of viral DNA must not be interpreted as an infection, since its detection does not determine infectivity. In spite of that, the knowledge of virus contamination is an important step towards linking fomites to infection risk, being more relevant in infections with agents with high environmental resistance. Parvoviruses such as AMDV or PCV-2 are well known to be able to survive a broad range or temperatures and different disinfectants [4,5,22,23]. Consequently, the detection of DNA of such viruses in the environment of infected farms should be considered as a real risk due to the probability of virions maintaining their viability.

To our knowledge, this is the first investigation using qPCR for evaluating the protection of different PPE against viruses under field conditions. The identification of viral DNA in PPE was consistent with the high level of surface contamination detected on the studied farms, and suggests an exposure to virus during visits despite the absence of direct contact with animals. Viral load detected in external coveralls and bootcovers was low, and lower than in the floor, cages, or walls of farms, probably because the short exposure time during the visit only leads to a low viral contamination level of PPE. Contaminations of coveralls with no direct contact with animals was already reported for AMDV [19] and PCV-2 has been detected in air samples [24]. These results may aid to make visible that only the mere presence in a contaminated environment is enough for detect contamination in clothes and thus, constitute a potential risk of transmission for certain viruses.

Regarding coveralls, visitors in the non-certifed PPE group presented a high contamination of internal elements despite the fact that these were covered by non-certified external elements. Thus, non-certified elements seem not to be sufficient to preserve visitors from viral contamination of base-layer clothes during visits to farms. However, in the certified group, one sample from street clothes tested positive for each farm, involving a different visitor, despite external C-Coverall having been used. Since there was no rupture of coveralls, samples prior to visit tested negative, and certified coveralls were used in this group, the presence of viral DNA is probably due to an accidental contamination during the undressing of PPE. Contamination of skin and clothes is reported to be frequent during the process of PPE undressing in hospitals [25], and considering the low viral load detected in the street clothes, this must be the most likely cause of DNA contamination here. In field conditions, standardized protocols for undressing PPE are not usually considered. For this reason, in this study visitors were not advised to follow any special protocol in order to emulate the typical situations in farms. These results suggest that contamination of base-layer clothes may occur, even using certified coveralls, if proper care during the undressing of coveralls is not taken. Besides, the fact of that, in our study, two different visitors were involved in the contamination indicates that this was not consequence of a systematic mistake of one person. On the contrary, this fact points out that this kind of contaminations are expected to be often and it is emphasized the need of disposing of valid protocol for undressing PPE in order to avoid potential risks. There are no abundant reports on the contamination during removal of PPE in farms; however, in health care personnel from hospitals, a frequent contamination of skin and clothing has been reported, especially during removal of gloves [25]. Basing on our results, personnel contamination in farms is expected to occur, thus it should be considered the implementation of recommendations for removal of PPE similarly to those described by the Center for Disease Control and Prevention or the guidelines already recommended in hospitals [26,27]. Besides, educational interventions to make awake farmers and veterinarians about the risk would be valuable to achieve a better level of biosecurity.

Shoe covers and street shoes samples in the non-certified PPE group were highly contaminated when compared with those in the certified PPE group, suggesting a different effectiveness between S-Bootcovers and C-Bootcovers to protect from contamination. After the visits, all S-Bootcovers were perforated, so this is likely the source of contamination of internal covers. These materials are mainly designed to be used with non-wrinkled surfaces; since these types of surfaces are not common in farm environments, small breaks can appear in the bootcovers, as found in this study. In addition, in the non-certified PPE group, a higher mean of number of copies per sample was detected in shoe covers than in S-Bootcovers, despite the fact that shoe covers were worn under S-Bootcovers. This could be due to viral entrance through holes in S-Bootcovers and subsequent accumulation in the space between covers. Consequently, broken S-Bootcovers did not protect from contamination, having a negative accumulative effect of viral particles. Similarly, the positive sample of shoe covers in the certified-PPE group was detected in the visitor wearing a broken C-Bootcovers. Thus, after looking at the risk of rupture of these materials that clearly compromise the protective effect of these elements, it would be worthwhile to alternatively consider the use of rubber boots or other materials with resistance to abrasive surfaces and that may be easily decontaminated to be reused.

Wearing the hoods of C-Coveralls (certified PPE group) prevented hair contamination, in contrast to that observed in the non-certified PPE group. These results indicate that contamination of non-covered body parts is possible and suggest the need to use more adequate PPE or the establishment of routines such as having a shower after a visit or compulsory down times to prevent carry over of viruses [28,29].

Biosecurity measures constitute one of the pillars of the animal health strategy of the European Union in the recent years, under the motto “prevention is better than cure” [30]. A better control of the exposure to contaminations during visits is a clear component of this strategy as numerous contagious pathogens may easily spread from one farm to another. Safe removal of PPE and decontamination of PPE seem also crucial to avoid dissemination of diseases, since the risk of carrying viral particles outside the farm may exist. Our results are consistent with this fact and reinforce the idea of considering visits are among the potential risks for disease dissemination. Biosecurity in farms is currently achieved through a combination of both nationally legislated and voluntary on-farm measures. The European Animal Health Regulation emphasises the responsibility of farmers for applying preventive measures, including on-farm biosecurity, in order to control contagious diseases within the European Union [31]. Thus, the use of PPE for visitors is not compulsory and relies on farmers’ willingness, which may be variable. Most of the farmers are concerned about biosecurity and were aware of its importance in preventing and controlling diseases; nevertheless, biosecurity measures are sometimes inconsistently applied on commercial farms [28], or farmers can have a low intention to make visitors to use PPE [32]. Besides, the application of these elements is also influenced by their cost. Thus, when PPE is used, non-certified elements are usually chosen because they are cheaper, and farmers may expect a sufficiently effective protection from them. The present results show that non-certified PPE does not really confer effective protection against viral contamination of clothes. Moreover, it is possible that the use of such elements of biosecurity might give a false sensation of protection, increasing the chance of viral contamination. As a result, future work in education about the risk and in the rationalized use of PPE is needed. Following these principles, our results should serve to make evident the real risk of contamination for certain viruses even though no direct contact exists, which is a situation that seems to go easily unnoticed. Therefore, it is important both veterinarians and farmers to be awake that the use of suitable PPE together with correct protocols/routines for achieving effective protection is required, as it is shown that this risk of contamination may be expected to be high.

One major impediment to the application of these measures of control may be related to the costs of the use of PPE and molecular diagnosis. Nevertheless, it should be noted that the entrance of some infections in a naïve farm may have a disastrous impact in the economy. The main and the cheapest biosecurity measure for a farm has to be minimize visits to a reasonable low level. But when entries in a farm are not avoidable, the use of certified PPE should be highly considered despite its cost, because our results prove that non-certified PPE are a real risk for disease transmission. Overall, the application and cost of the establishment of these measures may be mitigated by a proactive investment in prevention in consonance with the animal strategies promoted by the European Union [30]. It may be expected that a change in some attitudes may have a good impact in the global biosecurity without applying expensive changes, since wrong practices are shown to be potentially high [32]. Thus, more expensive measures may be reserved to determined situations as a result of cost-benefit analysis and the assessment of the particular epidemiological risk of a given farm. The implementation of a certain level of molecular diagnosis may have also an educational value to increase the biosecurity levels. It has been shown that the limited examples of proven efficacies, combined with the lack of relevant education are potential reasons for infrequent or non-compliance to biosecurity measures [12]. Thus, the application of qPCR to environmental and PPE samples can facilitate veterinarians to educate farmers about the importance of biosecurity, providing an objective way to value these risks.

In any case, it seems necessary to make a reasoned approach to this question on the basis of designing adequate protocols. In this context, qPCR can be considered a useful tool for assessing PPE contamination as well as for designing and validating such protocols. Also, in the last years different efforts have been made to standardize sampling procedure as well as monitoring residual contaminations. The application of molecular diagnosis techniques to assess biosecurity risks is of great interest to monitor and analyze risks, in consonance with the challenges for the H2020 program, that include the design, development and deployment of new diagnostic tools and intervention strategies [33]. Cost of DNA-based methods may be a limitation for their routinely use, but they can be useful to validate biosecurity protocols and generalize such protocols to the population afterwards. Consequently, a future step in this work should include the economic assess of the implementation of these methods. In this study, we applied qPCR because of its robustness and availability; however, other molecular techniques could be implemented and evaluated like isothermal amplification that may provide advantages regarding simplicity and appropriateness for in-field use [34]. This kind of alternatives should be considered for a progressive optimization of the resources to assess contamination in PPE for in-field diagnosis.

Overall, a good protocol of biosecurity in farms must be easy to be applied in field conditions and accepted by farmers. According to our results, we propose that it should include, at least, the use of rubber boots or boots with reinforced soles that can easily resist wrinkled surfaces and be thoroughly decontaminated, the use of certified PPE, as well as procedures for decontamination and removal of PPE at the end of the visit, because it has been proved that these contaminations may occur. For this last step, the recommendations already established by health organizations to handle risks of contaminations in human medicine [26,27] may be taken in consideration to be incorporated to biosecurity measures in farms, as well as it should be recommended the use of showers to eliminate the contamination of non-covered parts. Also, in our opinion and with an advisable design, qPCR could be convenient to monitor biosecurity on farms. Therefore, a standard framework, similar to the Hazard Analysis and Critical Control Point programs that are widely applied in food safety, could be applied to biosecurity [35].

In conclusion, workers and personnel should use certified coveralls with barriers to infective agents and receive training in their proper use in order to effectively prevent viral contaminations on farms. Special care must be taken to avoid perforations of bootcovers, especially on abrasive surfaces. Although both studied farms presented different infections and different levels of environmental contamination, the results reported for both farms were consistent and indicate that the used protocol of DNA detection can be applied to diverse conditions. Additionally, these results may serve as a model to evaluate other biosecurity measures and PPE related to indirect transmission of infections. Consequently, qPCR can be considered a useful tool in the risk management of biosecurity programmes.

## Supporting information

S1 File Clothing models for certified and non-certified PPE groups. (PDF)
