# Supplementary material for: Molecular assessment of visitor personal protective equipment contamination with the Aleutian mink disease virus and porcine circovirus-2 in mink and porcine farms
Source: PLoS One. 2018 Aug 27;13(8):e0203144. doi: 10.1371/journal.pone.0203144 (PMC6110490; doi:10.1371/journal.pone.0203144)

Clothing model of certified PPE group

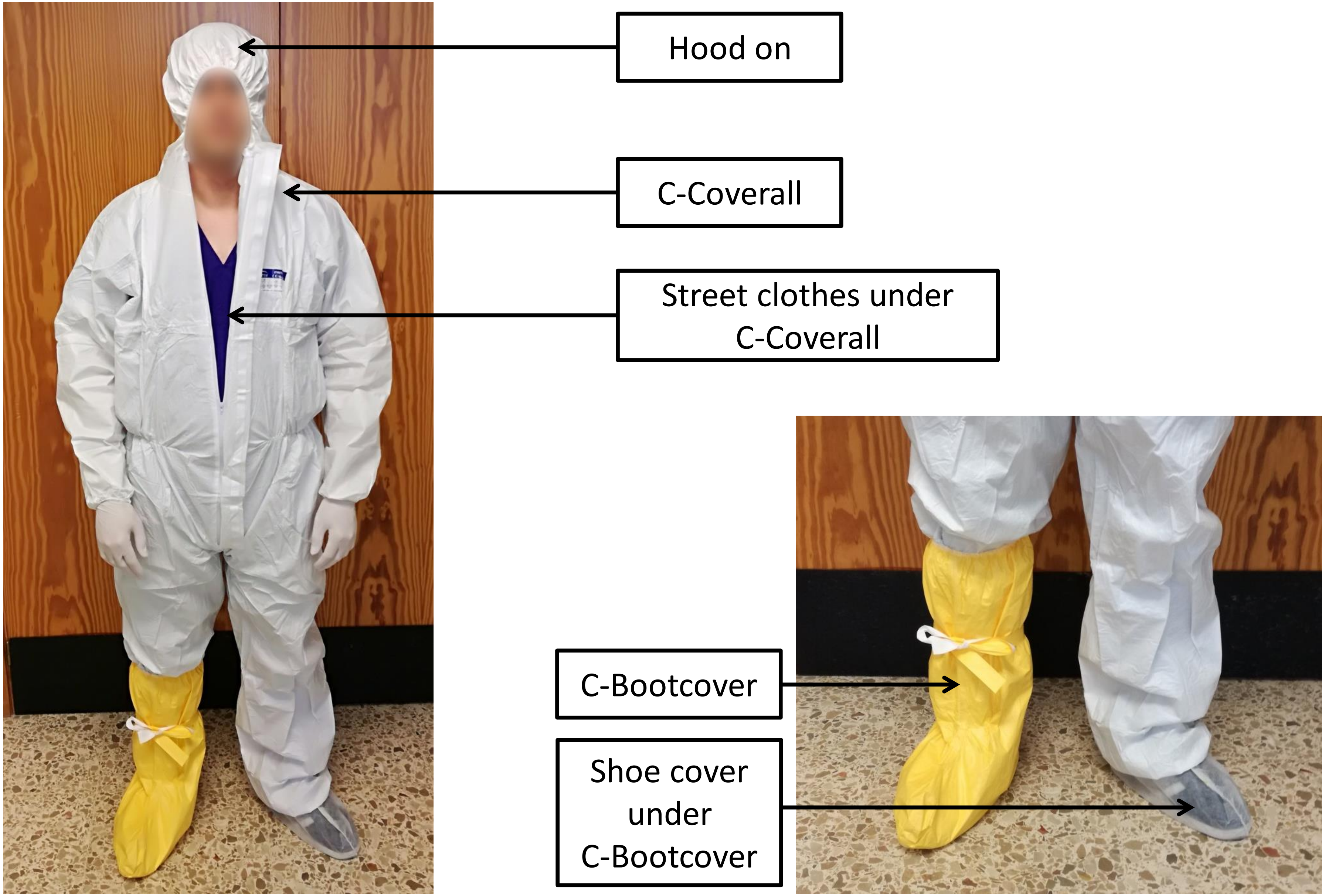

Clothing model of non-certified PPE group

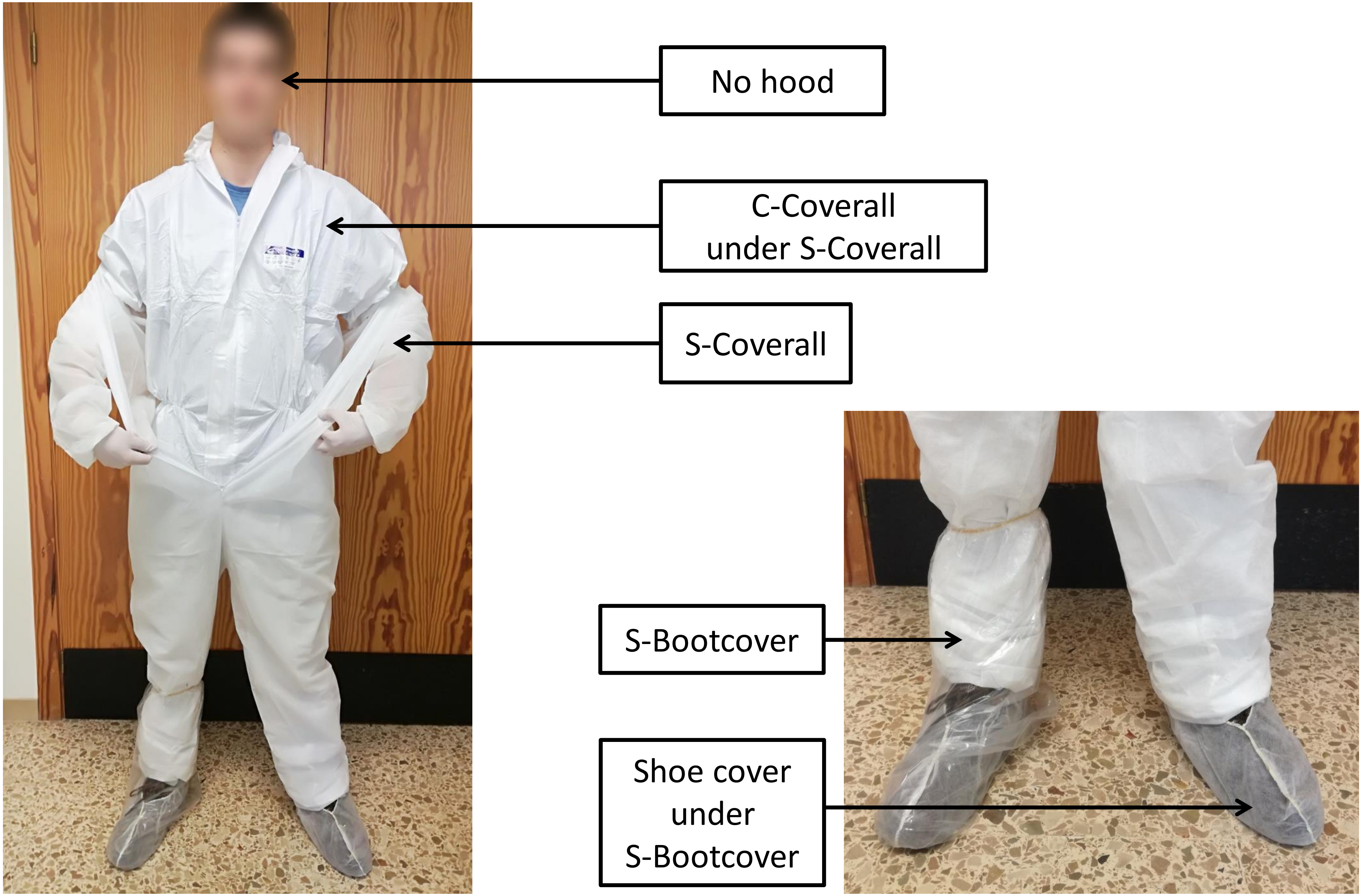

Supplement: S1 File — (PDF) [file pone.0203144.s001.pdf]
